# Structural Characterization of SARS-CoV-2: Where We Are, and Where We Need to Be

**DOI:** 10.3389/fmolb.2020.605236

**Published:** 2020-12-17

**Authors:** Giuseppina Mariano, Rebecca J. Farthing, Shamar L. M. Lale-Farjat, Julien R. C. Bergeron

**Affiliations:** ^1^Microbes in Health and Disease Theme, Newcastle University Biosciences Institute, Newcastle University, Newcastle upon Tyne, United Kingdom; ^2^Randall Centre for Cell and Molecular Biophysics, King’s College London, London, United Kingdom

**Keywords:** COVID19, SARS-CoV-2, coronavirus, structural biology, cryo-EM

## Abstract

Severe acute respiratory syndrome coronavirus 2 (SARS-CoV-2) has rapidly spread in humans in almost every country, causing the disease COVID-19. Since the start of the COVID-19 pandemic, research efforts have been strongly directed towards obtaining a full understanding of the biology of the viral infection, in order to develop a vaccine and therapeutic approaches. In particular, structural studies have allowed to comprehend the molecular basis underlying the role of many of the SARS-CoV-2 proteins, and to make rapid progress towards treatment and preventive therapeutics. Despite the great advances that have been provided by these studies, many knowledge gaps on the biology and molecular basis of SARS-CoV-2 infection still remain. Filling these gaps will be the key to tackle this pandemic, through development of effective treatments and specific vaccination strategies.

## Introduction

Recently, a novel Coronavirus (CoV), SARS-CoV-2, has emerged in China at the end of 2019 and quickly spread nearly to every country, causing a global pandemic. SARS-CoV-2 causes severe infections of the respiratory tract, with symptoms that range from dry cough, to fever and pneumonia, and has a high mortality rate (Benvenuto et al., 2020; Huang et al., 2020; Mousavizadeh and Ghasemi, 2020). As of mid-November 2020, it has infected an estimated 60 M people word-wide, and caused > 1.4M deaths (World Health Organisation, 2020). The virus is still endemic in most countries, and no vaccine or prophylactic drugs have been approved yet to prevent its spread. Two drugs (Remdesivir and Dexamethasone) have recently been approved in its treatment, but these only help alleviate symptoms in the most severe case (National Institute of Allergy, and Infectious Diseases (NIAID), 2020; The Recovery Collaborative Group, 2020).

Coronaviruses (CoVs) belong to the family of Coronaviridae. These are enveloped viruses that contain a positive, single-stranded RNA genome, which is packaged within a capsid. The capsid consists of the nucleocapsid protein N and this is further surrounded by a membrane, that contains three proteins: the membrane protein (M) and the envelope protein (E), which are involved in the virus budding process, and the spike glycoprotein (S), which is a key player in binding host receptor and mediating membrane fusion and virus entry into host cells (Li, 2016; Cai et al., 2020).

CoVs can be divided in four genera, *Alphacoronavirus*, *Betacoronavirus*, *Gammacoronavirus*, and, *Deltacoronavirus* (Woo et al., 2012; Cui et al., 2019). *Alpha* and *Betacoronaviruses* more commonly cause infections in humans and mammal (Tang et al., 2015) and in particular, *Betacoronaviruses* include severe acute respiratory syndrome coronavirus (SARS-CoV) and Middle East respiratory syndrome coronavirus (MERS-CoV), which caused previous pandemics in 2002 and 2012, respectively (Snijder et al., 2003; Chan et al., 2015), and the newly emerged virus SARS-CoV-2. *Gammacoronavirus*, and *Deltacoronavirus* instead prevalently infect birds and fish, but some instances were also found to infect mammals (Woo et al., 2010). The main distinctive characteristic between the 4 genera is the presence of the non-structural protein Nsp1 in *Alpha* and *Betacoronaviruses*, with Nsp1 displaying distinct protein size and sequence features between these two genera (King et al., 2012). Conversely, a Nsp1 counterpart has not been reported in *Gammacoronavirus*, and *Deltacoronavirus* (King et al., 2012). Furthermore, *Alphacoronaviruses* exclusively possess a common accessory gene which encodes for the multi-spanning alphacoronavirus membrane protein (αmp) (King et al., 2012). Different types of *Alphacoronaviruses* can possess a different number of copies of this accessory gene (King et al., 2012). Within each genus, different types of CoVs will be equipped with different types of accessory genes, determining the distinctive host-range, virulence and mortality rate of each CoV subtype.

SARS-CoV and MERS-CoV are highly virulent and caused global pandemics in 2002 and 2012, respectively, with high mortality rates (∼10% for SARS-CoV and ∼36% for MERS-CoV) (Rota et al., 2003; de Groot et al., 2013; Li, 2016). Similarly, SARS-CoV-2 shows high mortality rate (reported globally as ∼ 3.8%) (World Health Organisation, 2020). SARS-CoV-2 additionally shows a higher infection rate compared to the closely related SARS-CoV (Benvenuto et al., 2020; Huang et al., 2020; Mousavizadeh and Ghasemi, 2020).

**FIGURE 1:**
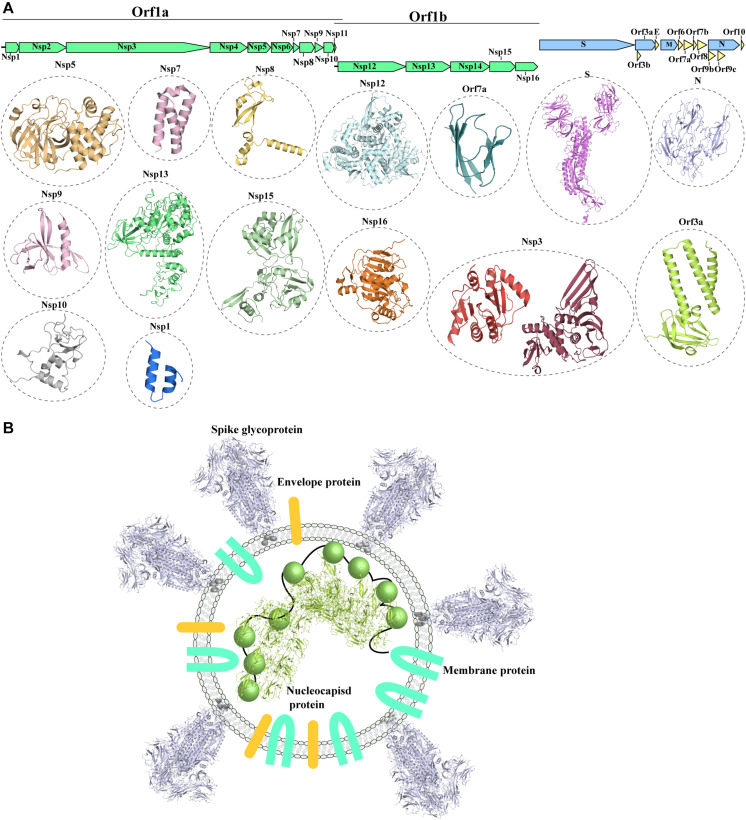
Known structures for SARS-CoV-2 proteins. **(A)** Schematic representation of genomic organization of SARS-CoV-2. Structural proteins are shown in pale blue, non-structural proteins are shown in green and accessory proteins are represented in pale yellow. Where available, a cartoon representation of the 3D structure for each protein is shown. 3D structure representations are based on PDBIDs shown in Table 1, only individual monomers are shown. **(B)** Schematic representation of an assembled SARS-CoV2 virus. The structural S glycoprotein is depicted through the use of a cartoon representation of its molecular structure (PDBID: 6VXX). E and M proteins are depicted with colored shapes. The nucleocapsid protein binding to viral RNA is represented by a cartoon representation of the molecular structure of its N-terminal domain (PDBID: 6M3M), while the C-terminal domain, whose structure is not available, is depicted with a colored sphere.

The SARS-CoV-2 genome (Wu F. et al., 2020) shows a similar organization to other CoVs. The positive-stranded RNA genome presents a 5′-cap and a 3′-poly-A tail (Figure 1A), allowing its translation from the host translation machinery. Similarly to other CoVs, at the 5′-end of SARS-CoV-2 a frameshift between two Orfs, Orf1a and Orf1b, allows the production of two polypeptides that are then proteolytically processed to produce 16 non-structural proteins (Nsp1-16) (Mousavizadeh and Ghasemi, 2020; Figure 1A), which are involved in various processes of the virus infection cycle (Gordon et al., 2020). At the 3′-end the structural proteins S (spike glycoprotein), N (nucleocapsid protein), M (membrane protein) and E (Envelope protein) are encoded. The nucleocapsid protein binds to the viral genome, aiding its packing against the internal surface of the envelope. The viral envelope is instead constituted of the S, M and E proteins (Paules et al., 2020; Figure 1B).

In addition to the 4 structural protein, the 3’-end also encodes nine accessory proteins (Orf3a, Orf3b, Orf6, Orf7a, Orf7b, Orf8, Orf9b, Orf9c, Orf10) (Figure 1A; Gordon et al., 2020). Accessory proteins were suggested to play an important role in virulence and host interaction in other coronaviruses (Liu et al., 2014). Whilst structural and non-structural proteins are shared amongst coronaviruses, the accessory proteins do not show highly similar distribution with other coronaviruses, with the exception of SARS-CoV (Liu et al., 2014). However, despite the close phylogenetical relationship between SARS-CoV and SARS-CoV-2 and their similar genomic organization, accessory proteins show decreased conservation, detectable both in lower sequence similarity between shared accessory proteins and variable content of accessory proteins between the two viruses (Table 1; Wu A. et al., 2020).

**TABLE 1:** Summary of available PDB structures of SARS-CoV-2 proteins.

Protein name	Function	PDB-IDs	Ligand	References	Sequence % ID SARS CoV
Nsp1	Inhibits translation machinery and production of immune defense factors	6ZLW	Ribosomal complex	Thoms et al., 2020	91%
		6ZM7	Ribosomal complex	Thoms et al., 2020	
		6ZME	Ribosomal complex	Thoms et al., 2020	
		6ZMI	Ribosomal complex	Thoms et al., 2020	
		6ZM0	Ribosomal complex	Thoms et al., 2020	
		6ZMT	Ribosomal complex	Thoms et al., 2020	
		6ZN5	Ribosomal complex	Thoms et al., 2020	
		6Z0N	Ribosomal complex	Thoms et al., 2020	
		6ZP4	Ribosomal complex	Thoms et al., 2020	
		6Z0J	Ribosomal complex	Schubert et al., 2020	
Nsp2	Putative role in apoptosis induction	–	–	–	83%
Nsp3	Involved in viral replication Nsp3-4-6 complex for ER modification and formation of DMVs Papain-like protease domain	6YWK	HEPES	–	87%
		6YWL	ADP-ribose	–	
		6YWM	MES	–	
		6W6Y	AMP	–	
		6WUU	Peptide inhibitor VIR250 ubiquitin propargylamide	Rut et al., 2020	
		6XAA	ISG15 C-terminal Domain propargylamide	Fu et al., 2020	
		6XA9	ISG15	Fu et al., 2020	
			ADP ribose		
			MES		
		6W9C	–		
		6VXS	–	–	
		6W02	ADP ribose	–	
		6WCF	–	–	
		6WEN	Peptide inhibitor VIR251	–	
		6WEY	–	–	
		6W0J	–	–	
		6WRH	–	–	
		6WX4	–	–	
		6WZU	–	–	
		6XG3	–	–	
Nsp4	Nsp3-4-6 complex for ER modification and formation of DMVs	–	–		91%
Nsp5	Main protease	6W63	Inhibitor X77	–	99%
		6WTT	Inhibitor GC-376	Ma et al., 2020	
		6XCH	Leupeptin	–	
		6WQF	–	Kneller et al., 2020	
		7C8R	TG-0203770	–	
		7C8T	TG-0205221	–	
		6WNP	Boceprevir	–	
		6WTK	Feline coronavirus drug		
		6WTM	Feline coronavirus drug	–	
		6WTJ	Feline coronavirus drug	–	
		6XB1	–	–	
		6XB0	–	–	
		6XB2	–	–	
		7BRR	GC376	–	
		7BR0	–	–	
		7BRP	Boceprevir	–	
		6M03	–	–	
		6XKF	–	–	
		6XHM	7V-[(2S)-l-({(2S,3S)-3,4-dihydroxy-1-[(3S)-2-oxopyrrolidin-3-yl] butan-2-yl}amino)-4-methyl-1-oxopentan-2-yl]-4-methoxy-1H-indole-2-carboxamide		
		6XHU	–	–	
		6XFN	UAW243	–	
		6XBG	UAW246	–	
		6XBH	UAW247	–	
		6XBI	UAW248	–	
		6X0A	–	–	
		6XMK	Inhibitor 7J	–	
		6YZ6	Leupeptin	–	
		6XA4	UAW241	–	
		7BQY	Inhibitor N3	–	
		6LU7	Inhibitor N3	–	
		6Z2E	Biotin-PEG(4)-Abu-Tle-Leu-Gln-vinylsulfone		
		6XKH	–		
		7BUY	Carmofur	Jin et al., 2020a	
		6M2N	–	–	
		6M2Q	–		
		6Y2G	Alpha-ketoamide 13b	Jin et al., 2020b	
		6Y2E	–	–	
		6YB7	–		
		6Y2F	Alpha-ketoamide 13b	Jin et al., 2020b	
		6LZE	Inhibitor 11b	Zhang et al., 2020b	
		6M0K	Inhibitor 11b	Zhang et al., 2020b	
		6Y84	–	–	
		7C8U	GC376	–	
		6YVA	mISG15	Zhang et al., 2020b	
		6YVF	AZD6482	–	
		6YT8	Pyrithione zinc	Dai et al., 2020	
		6YNQ	AZD6482	Dai et al., 2020	
Nsp6	Nsp3-4-6 complex for ER modification and formation of DMVs	–	–	–	95%
Nsp7	Part of the replication-transcription complex, involved in virus replication	7BTF	Nsp8, Nsp12	Gao et al., 2020	100%
		6M71	Nsp8, Nsp12	Gao et al., 2020	
		7BV1	Nsp8, Nsp12	Yin et al., 2020	
		7BV2	Nsp8, Nsp12, Remdesivir	Yin et al., 2020	
			Nsp8, Nsp12		
		7BW4	Nsp8, Nsp12	Peng et al., 2020	
		6YYT	Nsp8	Hillen et al., 2020	
		6M5I	Nsp8 C-term	–	
		6WIQ	Nsp8 C-term	–	
		6WQD	Nsp8 C-term	–	
		6WTC	Nsp8 C-term	–	
		6XIP	Nsp8, Nsp12	–	
		6X2G	Nsp8, Nsp12	–	
		7C2K	Nsp8, Nsp12	Wang et al., 2020a	
		7BZF	Nsp8, Nsp12	Wang et al., 2020a	
		6YHU	Nsp8	–	
		6XEZ	Nsp8, Nsp12, Nsp13	Chen et al., 2020	
		6XQB	Nsp8, Nsp12	Shi W. et al., 2020	
Nsp8	Part of the replication-transcription complex, involved in virus replication	7BTF	Nsp7, Nsp12	Gao et al., 2020	99%
		6M71	Nsp8, Nsp12	Gao et al., 2020	
		7BV1	Nsp7, Nsp12	Yin et al., 2020	
		7BV2	Nsp7, Nsp12, Remdesivir	Yin et al., 2020	
			Nsp7, Nsp12		
		7BW4	Nsp7, Nsp12	Peng et al., 2020	
		6YYT	Nsp7	Hillen et al., 2020	
		6M5I	Nsp7	–	
		6WIQ	Nsp7	–	
		6WQD	Nsp7	–	
		6WTC	Nsp7	–	
		6XIP	Nsp7, Nsp12	–	
		6X2G	Nsp7, Nsp12	–	
		7C2K	Nsp7, Nsp12	Wang et al., 2020a	
		7BZF	Nsp7, Nsp12	Wang et al., 2020a	
		6YHU	Nsp7	–	
		6XEZ	Nsp7, Nsp12, Nsp13	Chen et al., 2020	
		6XQB	Nsp7, Nsp12	Shi W. et al., 2020	
Nsp9	RNA replicase	6W9Q	Main protease-derived peptide		98%
		6W4B	–	–	
		6WXD	–	–	
Nsp10	Modulates Nsp14 and Nsp16 activities Nsp10-Nsp11-Nsp14-Nsp16-complex	7C2I	Nsp16, SAM	–	99%
		7C2J	Nsp16, SAM	–	
		6W61	Nsp16, SAM	–	
		6XKM	Nsp16, SAM	–	
		6WJT	Nsp16, *S*-Adenosyl-L-	–	
			Homocysteine		
		6WVN	Nsp16, *S-*Adenosylmethionine		
		6WQ3	Nsp16, *S-*Adenosylmethionine, 7-methyl-GpppA		
		6WKS	Nsp16, *S-* Adenosylmethionine, 7-methyl-GpppA		
		6WRZ	Nsp16, *S-* Adenosylmethionine, 7-methyl-GpppA		
		6ZCT	Nsp16, Sinefungin	–	
		6YZ1	Nsp16, Sinefungin	–	
		6WKQ	Nsp16	–	
		7BQ7	Nsp16, *S-*adenosylmethionine		
		6W75	Nsp16		
		6W4H	Nsp16, *S-*adenosylmethionine	Rosas-Lemus et al., 2020	
Nsp11	–	–	–		92%
Nsp12	Part of the replication-transcription complex, involved in virus replication	7BTF	Nsp7, Nsp8	Gao et al., 2020	98%
		6M71	Nsp7, Nsp8	Gao et al., 2020	
		7BV1	Nsp7, Nsp8	Yin et al., 2020	
		7BV2	Nsp7, Nsp8	Yin et al., 2020	
		7BW4	Nsp7, Nsp8	–	
		6YYT	Nsp7, Nsp8	Peng et al., 2020	
		6X2G	Nsp7,Nsp8	–	
		7C2K	Nsp7, Nsp8	Wang et al., 2020a	
		7BZF	Nsp7,Nsp8	Wang et al., 2020a	
		6XEZ	Nsp7, Nsp8, Nsp13	Chen et al., 2020	
		6XQB	Nsp7, Nsp8	Shi W. et al., 2020	
Nsp13	Helicase, part of the RNA polymerase complex, involved in virus replication	6XEZ	Nsp7, Nsp8, Nsp12	Chen et al., 2020	100%
Nsp14	Exonuclease activity and N7-MTase activity. Nsp10-Nsp11-Nsp14-Nsp16 complex	–	–	–	99%
Nsp15	RNA endonuclease	6VWW	–	Kim et al., 2020	96%
		6WLC	Uridine-5′-	Kim et al., 2020	
		6W01	Monophosphate Citrate	Kim et al., 2020	
		6WXC	Tipiracil	–	
		6XDH	–	–	
		6X41	3’-uridinemonophosphate	–	
		6X1B	GpU	–	
Nsp16	SAM-dependent, 2′-*O*- Methyltransferase/Complex with Nsp10 Nsp10-Nsp11-Nsp14-Nsp16 complex	7C2I	Nsp10, SAM	–	98%
		7C2J	Nsp10, SAM		
		6W61	Nsp10, SAM		
		6XKM	Nsp10, SAM		
		6WJT	Nsp10, *S*-Adenosyl-L- Homocysteine		
		6WVN	Nsp10, *S*-Adenosylmethionine		
		6WQ3	Nsp10, *S-*Adenosylmethionine, 7-methyl-GpppA		
		6WKS	-		
			Nsp10, *S-*		
		6WRZ	Adenosylmethionine, 7-methyl-GpppA		
		6ZCT	Nsp10, Sinefungin	–	
		6YZ1	Nsp10, Sinefungin	–	
		6WKQ	Nsp10	–	
		7BQ7	Nsp10, *S-*		
		6W75	adenosylmethionine		
			Nsp10,		
		6W4H	*S*-adenosylmethionine	Rosas-Lemus et al., 2020	
Spike (S)		6ZGH	–	–	87%
glycoprotein		6ZER	EY6A Fab	–	
		6VW1	ACE2	Shang et al., 2020	
		6WPS	S309 Fab	Pinto et al., 2020	
		6WPT	S309 Fab	Pinto et al., 2020	
		6VSB	–	Wrapp et al., 2020	
		6VXX	–	Walls et al., 2020	
		6VYB	–	Walls et al., 2020	
		6X6P	–	Herrera et al., 2020	
		7C01	CB6 antibody	Shi R. et al., 2020	
		7BWJ	P2B-2F6 Fab	Ju et al., 2020	
		7BYR	BD23-Fab	Cao et al., 2020	
		6M17	ACE2-B0AT1	Yan et al., 2020	
		6M0J	ACE2	Lan et al., 2020	
		6LZG	ACE2	Wang et al., 2020b	
		6LVN	–	–	
		6M1V	–	–	
		6ZGG	–	Xia et al., 2020	
		6LXT	–		
		6YM0	CR3022 Fab	Huo et al., 2020	
		6YOR	CR3022 Fab	Huo et al., 2020	
		6YLA	CR3022 Fab	Yuan et al., 2020b	
		6W41	CR3022 Fab	Yuan et al., 2020a	
		6X2C	CC12.1	Henderson et al., 2020	
		6X2A	–	Henderson et al., 2020	
		6X2C	–	Henderson et al., 2020	
		6X2B	–	Henderson et al., 2020	
		6X29	–	–	
		6YZ5	–	Yuan et al., 2020a	
		6ZGE	–	Yuan et al., 2020a	
		6ZGI	CC12.1 Fab	Henderson et al., 2020	
		6XC4	CC12.3/CR3022 Fab	Henderson et al., 2020	
		6XC3	CC12.1 Fab	Hsieh et al., 2020	
		6XC2	C105 Fab	Chi et al., 2020	
		6XCN	C105 Fab	–	
		6XCM	Cv30 Fab	–	
		6XE1	–	Chi et al., 2020	
		6XKL	4A8 Fab	Cai et al., 2020	
		7C2L	–	Cai et al., 2020	
		6XR8	–	Barnes et al., 2020	
		6XRA	Neutralizing antibody Fab fragment C105, state2	Barnes et al., 2020	
		6XCN	Neutralizing antibody	Barnes et al., 2020	
			Fab fragment C105, state1	Hansen et al., 2020	
		6XCM	Neutralizing antibody		
			Fab fragment C105		
		6XCA	2 Neutralizing antibodies		
		6XDG			
Orf3a	NLRP3 inflammasome activation	6XDC		Kern et al., 2020	85%
Orf3b	–	–	–	–	10%
Envelope (E) protein	Envelope protein, involved in virus packaging				96%
Membrane (M) protein	Membrane protein, involved in virus packaging				96%
Orf6	Interferon antagonist				86%
Orf7a	Induces apoptosis		6W37		90%
Orf7b	–	–	–	–	84%
Orf8	–	–	–	–	45%
Orf9b	Interferon antagonist		6Z4U		85%
Orf9c	–	–	–	–	78%
Nucleocapsid (N) protein	RNA binding and packaging	6WZO	–	Ye et al., 2020	94%
		6WZQ		Ye et al., 2020	
		6VYO		–	
		6ZCO		–	
		6YUN		–	
		6YI3		–	
		7C22		–	
		6M3M		Kang et al., 2020	
		6WJI		–	
		6WKP		–	
Orf10	–	–	–	–	N/A

Since the release of SARS-CoV-2 genome sequence, a significant effort has been directed to the investigation of the molecular mechanism of SARS-CoV-2 infection. In particular, many studies have focused on the structural characterization of SARS-CoV-2 structural, accessory and non-structural proteins (Figure 1A and Table 1) in order to elucidate in detail their involvement in the various steps of the infection process and to explore their role as effective molecular targets for therapy development.

The aim of this review is to summarize the progress towards the structural determination of SARS-CoV-2 proteins, but also to highlight the challenges that still remain and areas deserving further investigations. In particular, a more complete understanding of the network of interactions between the viral proteins, and how they cooperate in the crucial steps of the virus infection, will allow a comprehensive understanding of the infection mechanism and facilitate the identification of pivotal proteins and processes that can be exploited for structure-guided drug and vaccine design.

## Structural Proteins

### The Spike Glycoprotein

**FIGURE 2:**
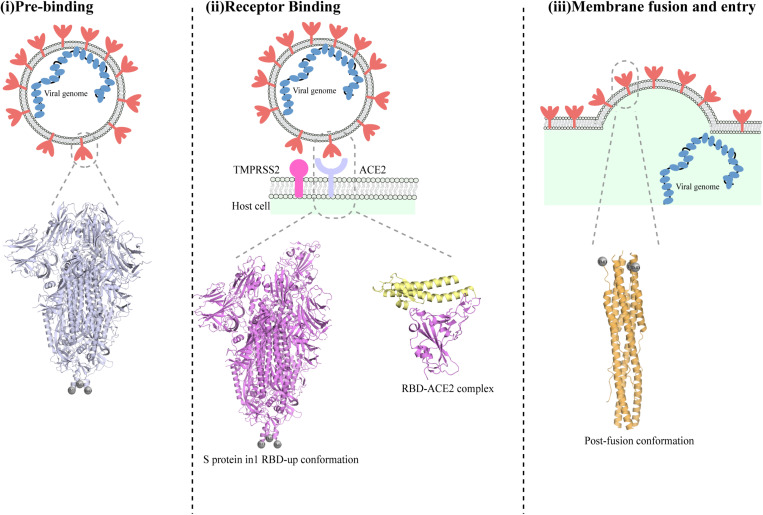
Different conformations of the SARS-CoV-2 spike protein S. Schematic representation of the stages of SARS-CoV-2 fusion to host cells membrane. The S trimer is exposed on the envelope of assembled viruses in its closed conformation **(i)**. Below the schematic, a cartoon representation of the SARS-CoV-2 protein in its pre-fusion closed conformation (PDBID:6vxx) is shown. Cleavage by furin allows exposure of RBDs in their ‘up’ conformation and binding to ACE2 receptor **(ii)**. The determined molecular structure of the S protein with one RBD open (PDBID:6vyb) is shown below. Following binding to ACE2 and further cleavage from TMPRSS2, additional conformational changes occurs that ultimately lead to the post-fusion conformation of the S protein and cause fusion between viral and host membrane **(iii)**. A cartoon representation of the structure of the post-fusion SARS-CoV-2 trimer is shown below the schematic in part **(iii)**.

The first step in SARS-CoV-2 infection is the invasion of a host cell, a process that is mediated by the spike (S) glycoprotein (Figure 2; Tortorici and Veesler, 2019). The S protein is a glycosylated type I membrane protein that consists of two subunits, S_1_ and S_2._ The S protein exists in a trimeric pre-fusion form that is later cleaved by a host furin protease into the two subunits S_1_ and S_2_ (Bosch et al., 2003). The N-terminal S_1_ subunit contains the receptor-binding domain (RBD), which mediated binding to the host cell receptor, namely the angiotensin converting enzyme 2 (ACE2) for both SARS-CoV and SARS-CoV-2 (Bosch et al., 2003; Hoffmann et al., 2020b). Binding of RBD to ACE2, followed by additional cleavage of the S_2_ subunit at a second specific site by the host serine protease TMPRSS2, are fundamental to trigger the disassociation between S_1_ and S_2_, leading to the conformational changes in S_2_ that are responsible for the fusion of viral and host membranes and virus entry (Figure 2; Hoffmann et al., 2020b; Walls et al., 2020).

Given its pivotal role in host recognition and invasion, the S protein has become a major target for the design of drugs and vaccine. Indeed, since SARS-CoV-2 sudden emergence, numerous studies have reported the structure of the S protein in various states (pre-fusion, post-fusion, and/or in complex with ACE2). A summary of all the available S protein structures, with their correspondent PDB ID and study is available in Table 1.

Numerous studies have reported the structure of the SARS-CoV-2 S protein by cryo-EM (Table 1) (some of them published merely a few weeks after the genome sequence of the virus was obtained, a remarkable tour-de-force). Two different approaches were used: in the first reported structures, the transmembrane (TM) domain of the protein was missing, and the ectodomain of the S protein was stabilized by introducing two prolines at the C-terminal S_2_ fusion domain, which stabilize the S protein soluble domain (Walls et al., 2020; Wrapp et al., 2020). Despite employing a similar strategy to yield a stable soluble domain, Wrapp et al. (2020) reported the structure of the S protein soluble domain in a partially open state (with one exposed RBD), whereas *Walls et al.*, obtained a sample with multiple conformational states of the S protein. Through 3D classification, Walls et al. (2020) have successfully separated the two species and resolved the structure of the S protein soluble domain in a closed conformation and in its partially open state (with one exposed RBD). One representative structure for the S protein soluble domain in a closed state and in a partially open state (with one exposed RBD) are shown in Figure 2. More recently the structure of the full-length S protein has been solved using cryo-EM (Cai et al., 2020). Interestingly, Cai et al. (2020) observed that the full-length protein exists *in vitro* as a mixture of different pre-fusion and post-fusion forms.

In several studies, isolation of monoclonal or polyclonal antibodies from plasma from recovered COVID-19 patients has produced a plethora of potential neutralizing antibodies with diverse targeted epitopes (Chi et al., 2020; Liu et al., 2020; Piccoli et al., 2020; Robbiani et al., 2020). In particular, in a study that evaluated ∼600 plasma and serum samples obtained from symptomatic and asymptomatic patients, it emerged that the majority of the elicited antibodies specifically bind to the S trimer RBD, with a minority recognizing other sites of the S trimer or binding to quaternary epitopes (Chi et al., 2020; Liu et al., 2020; Piccoli et al., 2020). Through the determination of the structure of six mABs, Piccoli et al., were able to map sites within the RBD that are predominantly recognized by the analyzed neutralizing antibodies. The study found that the dominant neutralizing strategy adopted by these antibodies includes binding to the receptor binding motif (RBM) which is only exposed in the S trimer open state or binding to a second site, which is also exposed in the closed state (Piccoli et al., 2020).

Epitopes targeted by the isolated antibodies have been precisely identified through determination of the structure of the S protein-candidate antibody complex with cryo-EM and X-ray crystallography. Barnes et al. (2020) isolated polyclonal antibodies which bound to the RBDs in ‘up’ conformation, sterically blocking ACE2 binding. Chi et al. (2020) instead isolated antibodies that were able to bind to the N-terminal domain (NTD) of the S protein and displayed high potency. Liu et al., isolated and characterized the epitope of a group of monoclonal antibodies that can target both the RBD and the NTD. Among these monoclonal antibodies, the structure of the 2-43 Fab in complex with the S trimer highlighted that three copies of the 2-43 Fab bind to a quaternary epitope on the spike protein, which in part includes the RBD of two adjacent protomers, when they are in the closed conformation (Liu et al., 2020). Similarly, Tortorici et al. (2020) identified a potent monoclonal human antibody, S2M11, which forms electrostatic interactions with a quaternary epitope that encompasses two of the RBDs of a S trimer. Whilst S2M11 and 2-43 Fab recognize distinct quaternary epitopes (Liu et al., 2020; Tortorici et al., 2020), they ultimately lead to locking of the S trimer in a closed conformation, achieving inhibition of ACE2 binding and virus entry. The study by Tortorici et al. additionally identified the S2E12 antibody, which binds to the receptor binding motif and thus can only bind to S trimer in the open state. In both cases, S2M11 and S2E12 prevent binding of the S trimer with ACE2, through targeting of different epitopes and when used in combination display additive effects on SARS-CoV-2 neutralization (Tortorici et al., 2020).

Intriguingly, the X-ray structure of the antibody CR3022 in complex with the S trimer showed that CR3022 can bind to the pre-fusion trimer leading to its transition to a disordered and inactive state (Huo et al., 2020). A full summary of the structures of the S trimer in complex with candidate neutralizing antibodies is shown in Table 1. The increasing effort of isolating and characterizing the epitope recognized by S protein-targeting antibodies becomes highly relevant in the context of a study from Hansen et al., where the authors showed that the use of cocktails of antibodies targeting the S protein display highest rate of success and lowest probability to lead to development of SARS-CoV-2 escape mutants (Hansen et al., 2020). The cryo-EM structure of two candidate antibodies bound to S, showed that these bind different epitopes of the RBD and thereby offer high protection against SARS-CoV-2 (Hansen et al., 2020), without increasing the rate of escape mutants. A similar potential for using antibodies in combination was displayed by S2M11 and S2E12 antibodies, which showed to both bind the S protein RBD, without competing. S2M11 and S2E12 were tested against several variants of the S protein and whilst some of them showed decreased binding to S2M11, they did not affect the neutralizing activity of S2E12 (Tortorici et al., 2020). The evidence of successful combination of different and potent antibodies in cocktails with high neutralizing properties presented by Tortorici et al., and Hansen et al., becomes particularly relevant in the context of the prevalence of RBD-targeting antibodies elicited in the immune response and the high frequency of mutations rising in the RBD region in escape mutants of SARS-CoV-2 (Li Q. et al., 2020; Piccoli et al., 2020). With the same principles, gaining structural information on more S protein-candidate antibodies will allow to design more efficient therapeutic strategies, taking advantage of the best combination of antibodies that can bind multiple sites of SARS-CoV-2 without posing steric obstacles to their partners and, thus, allowing to develop treatments with a low probability to rise escape mutants of SARS-CoV-2.

The structure of the S protein pre-fusion trimer consists of the S trimer with all three molecules in the closed state (Cai et al., 2020; Walls et al., 2020) or with one RBD in the open state (hereinafter referred to as ‘open state’) (Walls et al., 2020; Wrapp et al., 2020; Figure 2 and Table 1). Despite the high similarity between the different structures of the pre-fusion conformations, several minor discrepancies have emerged (Cai et al., 2020). In particular, the proximal region to the fusion subunit was found to be disordered in pre-fusion ectodomains in their closed state and in the available open state structure. In contrast, the same region was fully ordered in the full-length S glycoprotein structure in the closed state (Cai et al., 2020). Considering the suggested role of the transition from ordered to disordered fold of this region in the conformational changes that lead to the switch from closed to fully open state (Cai et al., 2020), the inconsistencies observed between available structures of the closed state need to be further investigated.

In a subsequent report, the S trimer was purified in baculovirus-infected Hi5 insect cells, with the use of media supplemented with free fatty acids. In these conditions, the obtained cryo-EM structure highlighted the presence of a hydrophobic fatty acid binding pocket located at the RBD of the S glycoprotein, in a distal position compared to the ACE2 binding motif that displayed specific binding to linoleic acid (Toelzer et al., 2020). The authors further report that the molecular features involved in fatty acid binding are conserved in SARS-CoV and less extensively in other coronaviruses, including MERS-CoV. Considering some differences in the 3D conformations of S protein bound to linoleic acid compared to previously determined structures, it has been proposed that previous structure represent an ‘apo form’ of the S protein, in absence of a ligand. Binding to linoleic acid seems to switch the RBDs from loosely to tightly packed in the closed conformation, opening to the possibility to exploit this feature to inhibit ACE2 binding and virus spread (Toelzer et al., 2020). However, lipid-binding pocket was not reported from structural studies that focused on the S-trimers in the context of the virion membrane (Ke et al., 2020). One emerging feature from the numerous studies that focused on the S trimer structure points out that experimental and purification conditions tend to have a great influence on the predominant conformation of the S trimer. Therefore, the remaining uncertainty on the physiological relevance of S-trimer lipid binding ability and its putative predominance in certain S-trimer conformations requires further addressing. Remarkably, cryo-ET studies that have focused on the study of the S protein in its physiological context have highlighted that the number of S proteins per virion, as well as the ratio of pre- and post-fusion conformation of the S protein is dependent on the level of expression of ACE2 and TMPRSS2 in the infected cell line used (Ke et al., 2020; Klein et al., 2020).

The structure of the post-fusion S protein was also determined, consistently shown to be quite rigid across independently determined structures, with a central stranded coiled-coil and two six helix-bundle that contribute to the rigidity of the central coiled-coil (Cai et al., 2020; Xia et al., 2020; Figure 2).

The structure of the RBD-ACE2 complex has been reported in several studies, employing both X-ray crystallography and cryo-EM (Lan et al., 2020; Shang et al., 2020; Yan et al., 2020). These structures showed that the SARS-CoV-2 S protein binds to the ACE2 receptor, similarly, to the SARS-CoV S protein and provided a comprehensive map of the amino acids residues involved in the RBD-ACE2 interactions (Lan et al., 2020). Despite the overall similarity, the RBD of SARS-CoV and SARS-CoV-2 shows a higher degree of divergence compared to other proteins (Table 1; Walls et al., 2020). Indeed, the SARS-CoV-2 S protein shows unique amino acids substitutions that establish additional contacts with the ACE2 receptor, compared to SARS-CoV S protein (Lan et al., 2020).

To date great progress has been made into characterizing the interaction of the S protein with ACE2 receptor and how this mediates virus entry, whereas not much information is currently available on the details of S protein interaction with the proteases involved in its processing and activation.

More detailed information on the molecular basis of the S protein rearrangements that allow transition from pre- to post-fusion conformation were provided by the combination of cryo-ET and molecular dynamics simulations (Turoòová et al., 2020). This study showed that the stalk that connects the S protein ectodomain to its trans-membrane domain possesses three flexible hinges (Turoòová et al., 2020). The subtomogram averages also allowed to visualize a right-handed coiled-coil region at the top of the stalk region, which the authors speculate might be involved in conformational rearrangements that allow to transition from the pre- to the post-fusion states (Turoòová et al., 2020).

Human and lung and bronchial cells were observed to co-express ACE2, TMPRSS2 and furin (Lukassen et al., 2020) and consistently, the necessity for both proteases for the efficient activation and pathogenicity of SARS-CoV-2 was shown in several studies (Bestle et al., 2020; Hoffmann et al., 2020a).

TMPRSS2 protease activity was found to determine activation of fusion proteins in many viruses, including SARS-CoV, MERS CoV, influenza A virus and hepatitis C virus (Kido et al., 2008; Sakai et al., 2014; Esumi et al., 2015; Böttcher-Friebertshäuser, 2018).

In contrast to SARS-CoV, where the S_1_-S_2_ boundary is cleaved by Cathepsin L (Bosch et al., 2008), SARS-CoV-2 shows a furin cleavage site between the S_1_ and S_2_ subunit. A similar cleavage site is also found on the spike protein of MERS CoV (Millet and Whittaker, 2014).

It is speculated that furin-dependent cleavage between S_1_ and S_2_ could mediate conformational changes that ultimately lead to exposure and binding of the RBD to ACE2 and exposure of the TMPRSS2 cleavage site (Millet and Whittaker, 2015; Hoffmann et al., 2018). However, molecular details of the interaction between furin and the SARS-CoV-2 S protein are currently not available. Interestingly, a structure for human furin has been solved and exploited for designing of small protease inhibitors (Dahms et al., 2017). The availability of this structure could not only favor the design or repurposing of existing drugs, but it could aid to establish important amino acid positions for furin-S protein contacts and help to determine the consequences and importance of the furin-dependent cleavage on the S protein conformational changes.

TMPRSS2 is responsible for cleavage of the S_2_ subunit at a second site, which ultimately leads to production of a fusion protein that mediates fusion of virus and host membrane and virus entry. To date structure of TMPRSS2, either on its own or in complex with the SARS-CoV-2 S protein has not been determined. However, in a recent computational study the structure of TMPRSS2 was modeled, showing in detail the fold of TMPRSS2 domains, including its C-terminal catalytic domain (Hussain et al., 2020). The proposed S protein cleavage sites were employed for docking simulations that allowed to locate TMPRSS2 and S protein residues involved in the interaction, including those residues neighboring cleavage sites and the C-terminal catalytic domain of TMPRSS2 (Hussain et al., 2020). These results represent a starting point for the investigation of priming of S protein and the conformational changes that drive virus entry, especially considering the intrinsic difficulties of solving TMPRSS2 structure experimentally (Hussain et al., 2020). Nevertheless, further investigation, utilizing both experimental and computational methodologies, that takes into account also the novel findings in S protein full length structure (Cai et al., 2020) might confirm or implement this current model.

For SARS-CoV it has been reported that TMPRSS2 and human airway trypsin-like protease (HAT) cleave and prime the ACE2 receptor and this is fundamental to enhance infectivity of SARS-CoV (Heurich et al., 2014). Whether this process is crucial also for SARS-CoV-2 entry and whether it also involves the furin protease remains to be established. If both proteases play a multifaceted role in the virus entry, the hierarchy and interdependence of these processes and their molecular aspects has to be established in order to safely exploit them in drug and therapy design.

Interestingly, treatment with protease inhibitors in some studies has shown to be largely effective in inhibition of SARS-CoV-2 infection indicating that targeting of furin and TMPRSS2 activity might represent a promising route for treatment (Bestle et al., 2020; Hoffmann et al., 2020b) and further highlighting the necessity to determine of TMPRSS2- S protein and furin-S protein complexes, with the ultimate aim of providing alternative routes of inhibition for structure-guided therapeutic strategies.

Finally, none of the S protein structures in closed or open state have yielded defined densities for the trans-membrane domains (TMDs) (Figure 2) – see Box 1. Following conformational changes in the post-fusion trimers, the fusion peptide and the trans-membrane regions come in close proximity, thereby initiating the membrane fusion (Schroth-Diez et al., 2000; Schibli and Weissenhorn, 2004). In a study on SARS-CoV, systematic point mutations within the trans-membrane domain on the S protein has allowed to elucidate residues in these regions that are fundamental for successful virus entry (Corver et al., 2009). The authors of this work propose a model whereby, following transition to a post-fusion state, TMD’s tryptophan residues bind to the membrane, causing rearrangements of the TMD’s hydrophobic region and insertion in the membrane of its neighboring region. TMDs then oligomerise, ultimately leading to membrane curvature (Corver et al., 2009). This model provides a starting point in understanding the role of TMDs in the membrane fusion, albeit it requires experimental validation. Progress in this direction has been made by Ke et al., with the *in situ* resolution of the S trimer structure. Compared to other available structure, this study was able to obtain additional densities for TMDs proximal regions. The study further shows that the TMDs proximal regions act as hinge that allow bending on the surface of some S trimers (Ke et al., 2020). Nevertheless, the flexibility of this region still represents an obstacle in the full resolution of the membrane-associated region of the S trimer. Efforts to determine the TMD structure and placing this in context with the available molecular details of others S protein structures may lead to a more in depth understanding of virus entry process.

**BOX 1. SARS-CoV-2 membrane proteins and membrane-embedded domains.**

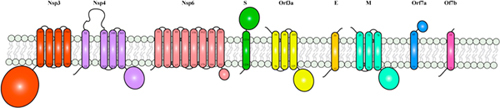

**Figure |** Schematic representation of SARS-CoV-2 membrane proteins. Trans-membrane helices were predicted using TMHMM Server v. 2.0. All membrane proteins were represented as monomers.

Membrane proteins exhibit significant challenges in their recombinant expression, purification, solubilisation and stability, which represent major obstacles in obtaining their structures. X-ray crystallography has a particularly low success rate with membrane proteins, as the use of detergents, protein stability and flexibility often prevents their crystallization (Rawson et al., 2016). In recent years, advances in cryo-EM have allowed to overcome some of these challenges and to increase the success rate of membrane proteins structure resolution. Detergent usage, whilst hindering preparation of cryo-grids, less frequently represents a problem in structure determination. Additional biochemical methods such as nanodiscs and amphipols, which helps with their solubilization but are not compatible with crystallization, have also contributed to successful cryo-EM analyses of membrane proteins. Nevertheless, heterogenicity of the protein sample and highly flexible regions still hamper the determination of high resolution molecular structures.

Within the protein repertoire of SARS-CoV-2, several proteins harbor one or more membrane domains. These include the structural proteins S, M and E, which are embedded in the virion membrane and permit virus fusion or budding. In addition, Nsp3-4-6, as well as the accessory proteins Orf3a, Orf7a and 7b, assemble at the plasma membrane or at the membrane of the Golgi complex or endoplasmic reticulum, during the various stages of the viral infection (von Brunn et al., 2007; Schaecher et al., 2008; Taylor et al., 2015). For Orf3a, studies conducted on SARS-CoV-2 have shown Orf3a mainly localizing at the plasma membrane (Kern et al., 2020; Ren et al., 2020).

Accordingly, cryo-EM was used in most studies focused on SARS CoV-2 proteins. One such example is represented by the two trans-membrane domains (TMDs) of the S protein. TMDs have been shown to mediate conformational changes that promotes virus entry, nevertheless despite the huge effort and the massive number of S protein structures that have been deposited during the past months (Table 1), near-atomic resolution determination of the TM region was not achieved in these analyses. Nonetheless, cryo-ET studies, in combination with molecular dynamics simulations yielded structures of the S protein in intact virions, and allowed to clearly distinguish the localization of TMDs of individual S copies (Turoòová et al., 2020). This study was not able to yield a high resolution structure of the TMDs, however, the authors were able to identify a right handed coiled-coil region at the top of a stalk region, which connects the TMDs to the ectodomain. The stalk region consists of three hinges, which provide flexibility to the S protein and lead the authors to speculate that it may contribute to drive the conformational changes that underlie the transition to a post-fusion conformation (Turoòová et al., 2020).

Similarly, the structure of the membrane channel Orf3a, determined by cryo-EM, has been reported recently. Orf3a formed dimeric and tetrameric ion channels, where tetrameric channels were found to be constituted by the juxtaposition of two dimers. Each dimer showed a novel fold, where the 3 TMHs of each protomer followed each other in a clockwise order, tracing an ellipse, ultimately forming a bifurcated channel, connected to the cytoplasm (Kern et al., 2020). Nonetheless, the structures of Nsp3, Nsp4, Nsp6, as well as E, M, and Orf7a and Orf7b, remain to be determined. Nsp3, Nsp4 and Nsp6 assemble at the endoplasmic reticulum, driving its modification into double membrane vesicles (DMVs) (Angelini et al., 2013; Hagemeijer et al., 2014). Numerous studies have reported the ability of Nsp3 to additionally interact with Nsp2, Orf3a, Orf7a and Orf9b (von Brunn et al., 2007; Neuman et al., 2008; Lei et al., 2018), which leads to the speculation that more proteins may either be involved in DMVs formation or might intervene at an earlier or later stage of the virus replication cycle to this process. Orf7a is localized at the Golgi-endoplasmic reticulum intermediary compartment, whereas reports on Orf3a localization have been contrasting, reporting this protein at the plasma membrane or Golgi complex membrane (Kern et al., 2020; Ren et al., 2020). In SARS CoV, Orf3a was found to interact with N, M and S (Tan, 2005), which are also localized at the Golgi-endoplasmic reticulum intermediary compartment. It is possible that Orf3a might play a ‘bridging’ role between the DMVs formation and later virus budding phase.

It is likely that homotypic and heterotypic interaction between TMDs will play a crucial role in formation of these complexes. It is therefore increasingly clear that much of the future understanding of SARS-CoV-2 replication cycle relies on the possibility to obtain near-atomic resolution structures of these putative complexes and to establish their network of interactions can underlying their function.

In addition to the structural details of the trans-membrane domains of the S protein trimer and its conformational changes, it still remains to establish whether other envelope proteins are involved in its conformational switches or stabilization. Indeed, cooperation of the S protein with other structural components N, E and M was shown to play a role during the membrane fusion step in other coronaviruses (Bosch et al., 2005). The lower variability of the S_2_ fusion subunit render this region an ideal target for development of inhibitors that may prevent virus entry and several inhibitors targeting this infection step have been identified (Walls et al., 2020; Xia et al., 2020). Consequently, additional steps need to be taken in order to determine the full structure of the S protein trimer by itself and in the context of the viral assembly, in relation to the other structural protein. These findings will shed light on additional residues involved in conformational changes within the S protein during membrane fusion and also reveal key residues involved into interactions between the S and other structural proteins, that are essential for the membrane fusion step to take place. Identification of these crucial amino acid positions may guide to design of inhibitors that prevent virus entry by targeting critical regions of the protein, less likely to mutate if the SARS-CoV-2 virus subsequently goes through genetic drift, as observed in other RNA viruses (Treanor, 2015).

### The Nucleocapsid, Membrane and Envelope Proteins

Severe acute respiratory syndrome coronavirus 2structural proteins also include the nucleocapsid protein N, the membrane protein (M) and the envelope protein (E) (Figure 1B; Paules et al., 2020). Unlike the S protein, structural information on these proteins is limited.

The nucleocapsid protein N plays a multifaceted role in the infection cycle of CoVs (McBride et al., 2014). In SARS-CoV, the N protein was reported to bind to, and package the viral RNA into ribonucleoprotein RNP complexes (Figure 1B). The packaged RNPs particles are located on the internal face of the viral membrane, forming a separate layer from the envelope proteins M, E and S (Figure 1B). Furthermore, RNPs localization could be aided by the interaction between N and the C-terminus of the M protein (Chang et al., 2014).

The architecture of the RNPs within newly assembled virions has been recently elucidated with the use of cryo-ET. The authors of this study observed the RNPs form a cylindrical, semi-circular assembly of 16 nm that shows a preferred orientation. Within this assembly, multiple and aligned copies of N proteins form pillar-shaped densities that are stacked in parallel. This assembly ultimately results in the formation in two juxtaposed curved walls (Klein et al., 2020).

Additionally, in SARS-CoV, the N protein is recruited at the replication-transcription complex by Nsp3 and thus, is thought to be involved in viral genome replication (Cong et al., 2020). In particular, this interaction seems to involve the N-terminal domains of Nsp3 and the N protein and its function is to guide the viral genome to the newly assembled replication complex (Hurst et al., 2013; Lei et al., 2018).

The membrane protein M is embedded in the viral membrane (Figure 1B), through three predicted transmembrane helices. Its role is to drive the assembly of new virions within the host cells (Masters, 2006; Neuman et al., 2011). Coronaviruses M proteins were shown to oligomerize at the membrane of Golgi-endoplasmic reticulum intermediary compartment (Stertz et al., 2007) and were shown to induce apoptosis (Tsoi et al., 2014). S, N and E proteins are then recruited through interaction with the M protein. Thus, the current model suggests that the M protein acts as a scaffolding platform that recruits the other structural proteins and promotes membrane curvature during virion budding (Schoeman and Fielding, 2019).

Finally, the envelope protein E presents one trans-membrane domain and shows oligomerization properties. Indeed it was reported that E protein can self-interact, through its trans-membrane domain, to form a ion channel (Pervushin et al., 2009). Interaction between the C-terminus of E and M proteins guides E recruitment to the Golgi-endoplasmic reticulum intermediary compartment, initiating virus budding into host cells (Corse and Machamer, 2003; Masters, 2006; Schoeman and Fielding, 2019).

Additionally, several studies have suggested that the envelope protein E can also establish interactions with the nucleocapsid protein N (Tseng et al., 2014; Schoeman and Fielding, 2019). Currently it is thought that this interaction takes place independently from M-N and M-E interactions and its necessity in the assembly and release of viral particles is still unclear (Schoeman and Fielding, 2019).

So far, high-resolution structures have been reported for the N protein, but not M or E. The domain organization of coronaviruses N protein includes a RNA binding N-terminal domain, a C-terminal domain involved in dimerization (Chang et al., 2009, 2014) and a disordered serine/arginine rich linker, which plays a regulative role (Wu et al., 2009; Chang et al., 2014).

The structure of the RNA-binding domain of SARS-CoV-2 N has been determined recently, identifying the binding site and proposing a thorough characterization of the electrostatic interactions that take place between the viral RNA and the N-terminal domain (Kang et al., 2020). An independent study has instead unveiled the structure of the N protein dimerization domain, demonstrating that this domain can dimerise in a structure that is highly conserved with SARS-CoV. Interestingly, the authors showed that inclusion of an additional linker region allowed formation of homo-tetramers (Ye et al., 2020).

In the case of the E protein, only low-resolution structural details are available. Indeed, the structure of the E protein from SARS-CoV protein in lyso-myristoyl phosphatidylglycerol (LMPG) micelles, has been determined through NMR. The monomer of E consists of one trans-membrane helix, with two additional middle and C-terminal helical segments (Surya et al., 2018). Following determination of the pentameric state of E through BN-PAGE, the authors exploited the monomeric structure to dock a pentameric model, where the E pentamer displays a α-helical bundle fold, where the C-terminal tails coil around each other (Surya et al., 2018).

Conversely, to date the molecular structure of M is unavailable. M, N and E proteins were all found to be indispensable for the proper virus assembly and release in SARS-CoV and other coronaviruses (Siu et al., 2008). Definition of the missing structural details will not only help to determine the folding of these protein on their own, but it will provide also a considerable enhancement in the attempt to obtain higher resolution structural and molecular details of the RNPs and their packaging against the envelope, including the elucidation of network and hierarchy of the interactions between N,M,S,E that leads to the final viral assembly.

## Non-Structural Proteins

Coronaviruses non-structural proteins are translated from the precursors proteins pp1a and pp1b. These precursors are processed by the pp1a-encoded proteases, Nsp3 and Nsp5, and the resulting non-structural proteins (Nsp1-16) are then essentials in viral replication, transcription and production of envelope proteins (Gordon et al., 2020; Mousavizadeh and Ghasemi, 2020).

### The Replication-Transcription Complex

Following virus entry, replication of SARS-CoV-2 genome is operated by the cooperation of several non-structural proteins (Mousavizadeh and Ghasemi, 2020). The core RNA-dependent RNA polymerase (RdRp) consists of the protein Nsp12, which is bound to a heterodimer of Nsp7 and Nsp8. A second Nsp8 molecule interacts with Nsp12 at a different site to the Nsp7-8 heterodimer. Several groups have determined the structure of the Nsp12-7-8 subcomplex (Table 1), underlining the high conservation of the structural features of this complex between SARS-CoV and SARS-CoV-2 (Kirchdoerfer and Ward, 2019; Gao et al., 2020; Peng et al., 2020; Romano et al., 2020; Yin et al., 2020).

Interestingly, the independent studies reported an additional N-terminal β-hairpin motif that was not reported for SARS-CoV Nsp12 (Kirchdoerfer and Ward, 2019; Gao et al., 2020; Peng et al., 2020; Romano et al., 2020; Yin et al., 2020). Furthermore, the contact sites between Nsp12 and Nsp7-8 were determined and it was demonstrated that Nsp7 mediated the majority of contacts between the heterodimer and Nsp12 and that the two Nsp8 copies of this complex have differential conformations that ensure their binding to the right Nsp12 site (Table 1; Gao et al., 2020; Peng et al., 2020; Romano et al., 2020; Yin et al., 2020). Peng et al. (2020) further show that existing amino acid substitutions in SARS-CoV-2 Nsp7-8-12 overall decrease the efficiency of the RNA-dependent RNA polymerase activity and the thermostability of these proteins. The RdRp is one of the most promising drug targets identified to date. Structural details of the mechanism of inhibition of the anti-viral drug Remdesivir have been determined (Yin et al., 2020). Remdesivir is a nucleotide analog that is able to access the conserved RdRp active site and terminates elongation of the viral RNA (Yin et al., 2020). Given the high conservation of the active residues of the RdRp, Remdesivir has the potential to be employed as a broad-spectrum antiviral drug for several coronaviruses infections (Yin et al., 2020). Additionally, another independent study has solved the structure of RdRp in a post-translocation conformation, highlighting that the flexible N-terminal region of Nsp8 undergoes conformational changes during DNA elongation and these movements are then transmitted to the Nsp7-Nsp8 heterodimer causing, in turn, its conformational re-arrangements (Shi W. et al., 2020). Nsp7-Nsp8 heterodimer rearrangements are concomitant with translocation and nucleotide addition and are suggested to play a regulatory role of the polymerase activity (Shi W. et al., 2020).

The Nsp13 helicase is also part of the replication-transcription complex (RTC). The structure of Nsp13 in complex with the core RdRp complex has been determined, highlighting important protein-protein contacts between Nsp13 and Nsp7, Nsp8 and Nsp12 (Table 1). In particular, the Nsp13 zinc-binding domain interacts with the N-terminal regions of the two copies of Nsp8 that are part of RdRp, and the residues involved in these interactions are highly conserved in *Betacoronaviruses* (Chen et al., 2020). Additionally, this study shows that the nucleic acid-binding pocket is located directly in front of the downstream tRNA (Chen et al., 2020).

The structural details obtained for the Nsp13-RdRp complex highlighted that RdRp translocates the RNA in a 3′ – > 5′ direction, whereas Nsp13 translocates it in the opposite direction (Chen et al., 2020). For this reason Chen et al., proposed a ‘backtracking’ model, typical of proof-reading activity of polymerases complexes, whereby the active site of Nsp13 is divided into two ‘pockets’ by a β-hairpin, and backwards movement of Nsp13 generates a single stranded 3′-RNA that extrudes from the secondary pocket of Nsp13 active site. The authors further suggest that this process, could aid proof-reading during synthesis of the viral genome (Chen et al., 2020). Finally, the structural details of Nsp13-RdRp complex allowed to determine the molecular details of binding between ADP-Mg^2+^ and the N-terminal nidovirus RdRp-associated nucleotidyltransferase (NiRAN) domain, albeit the physiological role of this activity remains unknown.

Despite the advances achieved so far in structural characterization of SARS-CoV-2, it still remains to be established how the interaction between Nsp13 and Nsp8 N-terminal region influence the regulatory role of the Nsp8 N-terminal region and the Nsp7-Nsp8 heterodimer. Additionally, an interaction between Nsp8 and Orf6 has been reported in SARS-CoV (Kumar et al., 2007), and is likely conserved in SARS-CoV-2 because of the high sequence similarity between the proteins in the different viruses. However, the functional role of this interaction is not known, and how Orf6 and, potentially, other accessory protein can influence viral replication remain to be investigated.

Despite these studies have broadened the understanding of the mechanism of SARS-CoV-2 RNA replication, many details still remain elusive. The understanding of how other Nsp proteins cooperate to achieve efficient RNA synthesis is a crucial step in order to fully and efficiently target this process.

Nsp15 is a RNA uridylate−specific endoribonuclease (NendoU) (Deng and Baker, 2018), and forms a double−ring hexamer in isolation, with the active sites situated at the top and bottom faces of this hexamer (Table 1; Kim et al., 2020). This assembly is conserved also in SARS-CoV and MERS CoV Nsp15 (Bhardwaj et al., 2008; Zhang et al., 2018; Kim et al., 2020).

Whilst it’s not clear what the role of Nsp15 is in viral replication, it has been shown to co-localize with the RdRp complex in MHV (Shi et al., 1999).

Nsp9 instead shows a dimeric fold (Table 1; Egloff et al., 2004; Littler et al., 2020). In SARS-CoV, Nsp9 dimerisation was shown to be necessary for viral replication (Miknis et al., 2009). Littler et al., additionally showed that Nsp9 has low affinity for RNA binding. Nsp9 was also shown to co-localize with Nsp7-8-10 in MHV (Bost et al., 2000) and an interaction with Nsp7 was reported in Porcine Reproductive and Respiratory Syndrome Virus (Chen et al., 2017). The precise role of Nsp9, and of its interaction with other proteins, isn’t currently known.

Another necessary step for viral RNA production is RNA processing and capping, which protects the viral RNA from being targeted by the host cells immune system (Wang Y.-N. et al., 2020). In SARS-CoV-2, and more generally in coronaviruses, this function is accomplished by the Nsp10-14-16 subcomplex.

Nsp14 shows both an exonuclease activity, performed by its N-terminal domain, and a N^7^-Methyltransferase (MTase) domain at its C-terminus. Concerted activity of domains accomplishes both proof-reading and capping of the viral RNA, consistent with reports of their importance for correct viral replication (Chen et al., 2009; Eckerle et al., 2010; Bouvet et al., 2014).

The structure of SARS-CoV-2 Nsp14 has not been determined yet, but molecular details of Nsp10-14 interactions and important amino acids involved in these contacts have been determined in SARS-CoV (Chen et al., 2009; Bouvet et al., 2014).

Nsp10 plays multiple roles in RNA capping: its interaction with Nsp14 stimulates the exonuclease activity by stabilizing the N-terminal domain (Chen et al., 2009). Nsp10 can additionally interact with Nsp16, forming a hetero-dimer (Table 1), with 7-methylguanine-triphosphate-adenosine (m7GppA)-specific, 2′-O-methyltransferase activity. Following addition of the m7GppA cap to the RNA, in presence of a methyl group donor, Nsp16 catalyses the methylation of the nucleotide adjacent to the cap, at the 2′-O position (Chen et al., 2011; Rosas-Lemus et al., 2020). This interaction is responsible for activation of Nsp16 activity and completion of the second RNA capping step (Bouvet et al., 2010). The structure of the Nsp10-16 complex in SARS-CoV-2 revealed the high similarity of this complex in SARS-CoV-2, SARS-CoV and MERS CoV, underlining its conserved role (Rosas-Lemus et al., 2020).

Intriguingly, while Nsp10 is required for the function of Nsp14 and Nsp16 (Bouvet et al., 2014; Rosas-Lemus et al., 2020), no intrinsic enzymatic activity for this protein has been identified. It is likely that Nsp10 fulfills its role by simply acting as a scaffold for the recruitment of Nsp14 and Nsp16 to the replication-transcription complex and by triggering their specific activity. It remains to determine whether Nsp10 can spatially and temporally coordinate the proof-reading and capping activities of Nsp14 and Nsp16, also in relation to the timings of RNA synthesis operated by the replication-transcription complex.

### Nsp3, Nsp4, Nsp6 Subcomplex

Nsp3 is the largest SARS-CoV-2 protein. In coronaviruses, Nsp3 comprises multiple domains, suggesting a pleiotropic role (Lei et al., 2018). The domain composition and sequence conservation of Nsp3 across the coronaviruses genera is variable (Neuman et al., 2011). Nsp3 encompasses one or two papain-like protease domains which are responsible for the cleavage of several Nsp proteins, including Nsp3 self-cleavage (Lei et al., 2018). Cleavage site and target specificity of papain-like protease domains in Nsp3 can greatly vary across coronaviruses (Lei et al., 2018). This is mainly due to the fact that whilst papain-like protease domain 2 is present in all coronaviruses, presence of papain-like protease domain 1 is retained in only some viruses, generating the differential cleavage specificity (Lei et al., 2018). Similarly to SARS-CoV and MERS CoV, SARS-CoV-2 only possess one papain-like protease domain that is responsible for the cleavage activity (Rut et al., 2020). Additionally, the papain-like protease cleaves poly-ubiquitin chains, hampering the host inflammation response, and impairs type I interferon response by interfering with ISG15 modifications (Box 2) (Lindner et al., 2005; Clementz et al., 2010).

**BOX 2. SARS-CoV-2 proteins hijacking the ubiquitin-proteasome pathway.**

The ubiquitin-proteasome system plays a central role in regulating eukaryotic cells homeostasis (Lecker et al., 2006), and influence many cellular processes, such as signaling, apoptosis, immunity and development (Gao and Luo, 2006; Randow and Lehner, 2009). It consists of several enzymes that, in a series of sequential reactions, promote the attachment of multiple ubiquitin molecules to cellular protein to be targeted for degradation (Lecker et al., 2006). Because of its versatility and ubiquity, the ubiquitin-proteasome system is frequently hijacked by viral proteins, for example to promote the degradation of key proteins involved in defense mechanisms (Gao and Luo, 2006; Randow and Lehner, 2009; Biard-Piechaczyk et al., 2012).

In SARS-CoV and SARS-CoV-2, Orf6 and Orf9b represent two such examples of proteins hijacking the ubiquitination pathway, to induce the degradation of interferon-induced antiviral proteins: Orf6 targets STAT1 (Frieman et al., 2007; Lei et al., 2020), a nuclear messenger that is transported to the nucleus through karyopherin α2 (KPNA2) and karyopherin ß1 (KPNB1), where it stimulates transcription of interferon stimulated genes and determines an antiviral state of the cell (McBride and Reich, 2003). Conversely, Orf9b induces the degradation of MAVS, TRAF3 and TRAF6 (Shi et al., 2014; Cheng et al., 2015; Sa Ribero et al., 2020). Mitochondrial antiviral signaling proteins (MAVS) are activated by retinoic acid-inducible gene I-like receptors (RIG-I) (Wu and Hur, 2015), which in response to detection of viral nucleic acids promotes oligomerisation of MAVS. MAVS form large filaments on the surface of mitochondria and recruit several members of the tumor necrosis factor receptor-associated factor (TRAF), including TRAF3 and TRAF6 (Saha et al., 2006; Liu et al., 2013). TRAF factors are E3 ligases which can lead to activation of IκB kinase ϵ and IKKα/β (Guo and Cheng, 2007). These factors in turn activate interferon regulatory factor (IRF) 3 and 7 and NF-κB, which are translocated to the nucleus, thereby inducing the expression of type I interferon and proinflammatory cytokines (Shi et al., 2015). However, the molecular details of how this is achieved are not currently understood, and accordingly, the structural basis for this activity has not been identified.

Furthermore, an interaction was also reported between the N protein and the p42 subunit of the proteasome complex, speculating that this interaction might inhibit proteasome functions (Wang et al., 2010). This in turn might reduce inflammation, by reducing antigen presentation.

However this remains largely speculative. Nonetheless, characterization of the p42-N complex may aid to understand how N binding to the proteasome can result in its inhibition and how this could benefit the viral replication cycle. It is also noteworthy that Orf10, exclusively found in SARS-CoV-2 (and not in SARS-CoV), has been shown to interact with the Cullin 2 (CUL2) RING E3 ligase complex, possibly hijacking its function. It is not known what the implications of this interaction is for virus replication, but a similar complex is recruited by other viral proteins, including HIV Vif, Adenovirus E4Orf6, EBV Bzlf and others (Mahon et al., 2014). In all cases, the virus exploits this to target an antiviral protein for proteasome degradation, and it is therefore likely the case also for Orf10. Identifying its target could potentially provide new avenues for antiviral treatment.

Finally, in recent years it has been identified that de- ubiquitination, together with the ubiquitin-proteasome pathway, also plays a role in protein turnover regulation. Accordingly, many viruses have been found to encode de-ubiquitinase enzymes, and these proteins play an important role in viral replication (Bailey-Elkin et al., 2017). In SARS-CoV-2, the papain-like protease domain of Nsp3 also shows de-ubiquitinase properties and leads to the removal of ISG15 from target proteins (Fu et al., 2020). Although the role of ISG15 in antiviral response is not completely understood, ISGylation was shown to enhance JAK/STAT-dependent antiviral response and to reduce virus-dependent IRF-3 degradation, thereby promoting the expression of type I interferon genes (Lu, 2006; Malakhova and Zhang, 2008). Nsp3-mediated ISG15 modifications removal could therefore result in suppression of IFN-I production and favor the progression of the viral infection.

Because of its broad and multi-step impact on the viral life cycle, one can speculate that the ubiquitin-proteasome system could be a promising target for the development of specific and/or wide spectrum antiviral drugs. However, a better structural understanding of the molecular details and contacts involved in viral and ubiquitin-proteasome system protein-protein interactions is required for such targeted drug development.

Several structures of the SARS-CoV-2 papain-like domain, alone or in complex with inhibitors, have been deposited in PDB (Table 1) but some are not published yet. These structures show that the papain-like protease domain has a homo-trimeric fold, conserved with its SARS-CoV and MERS CoV counterparts. Remarkably, one promising study has revealed the structure of the papain-like domain with GRL0617, a repurposed drug that can target both SARS-CoV and SARS-CoV-2. GRL0617 binds to the SARS-CoV-2 Nsp3 papain-like protease domain away from its catalytic site, but instead inhibits Nsp3 ability to interfere with ISG15 (Fu et al., 2020).

In an independent study the structure of the papain-like protease in complex with several inhibitors, which have been engineered to carry a vinylmethyl ester to increase their reactivity toward deubiquitinases enzymes, was determined (Rut et al., 2020). This approach yielded irreversible inhibitors that are specifically active against SARS-CoV and SARS-CoV-2, but not MERS-CoV (Rut et al., 2020). The structural data obtained provided the molecular basis of inhibition operated by these compounds and their potential in prevention of the viral infection (Lei et al., 2018; Rut et al., 2020), highlighting that the papain-like protease/inhibitors complexes adopt an overall very similar structure and that inhibitors binding takes place in proximity of the active site, thereby preventing substrate binding (Rut et al., 2020).

Furthermore, Nsp3 possesses two ubiquitin-like domains that fulfill distinct roles. The ubiquitin-like domain 1 is positioned at the N-terminal of the protein and it can mediate interaction with the N protein and with ssRNA (Cong et al., 2020). The role of the ubiquitin-like domain 2 is object of contrasting reports, with one study suggesting it plays a role in inhibiting IFN production, while a distinct study suggests it is involved in innate immune response (Frieman et al., 2009; Clementz et al., 2010).

Finally, Nsp3 proteins have also a macro domain that is involved in disruption of the expression of innate immunity genes (Fehr et al., 2016). Interestingly *in vitro* experiments have shown the ability of SARS-CoV Nsp3 macro domain to interact with Nsp12, however, the physiological relevance of this interaction in the virus life cycle remains unknown (Imbert et al., 2008). Several structures of the macro domain have been released on PDB, although are still not published (Table 1) showing high similarity to MERS CoV Nsp3 Macro domain (Lei et al., 2018).

Aside from its protease and Ubiquitin-like domains, Nsp3 is also responsible for initiation of the assembly of the replication-transcription complex in SARS-CoV, mouse hepatitis virus (MHV) and MERS-CoV-infected cells and therefore is essential for virus replication (Lei et al., 2018). Nsp3 was shown to be interacting with a number of other CoV proteins, including Nsp2, Nsp4, Nsp6, Orf3a, Orf7a and Orf9b (von Brunn et al., 2007; Neuman et al., 2008; Angelini et al., 2013; Hagemeijer et al., 2014; Lei et al., 2018), highlighting the central role of the multi-domain protein in the virus life cycle and thus, rendering it an attractive target for therapeutic development. In particular, Nsp3, Nsp4 and Nsp6 have been shown to form a complex that modifies the endoplasmic (ER) reticulum into double membrane vesicles (DMVs) (Angelini et al., 2013; Hagemeijer et al., 2014). These studies also reveal that the C-terminal domain of Nsp3 is sufficient for DMVs formation, and further identified fundamental residues in Nsp4 involved in DMVs formation (Angelini et al., 2013; Hagemeijer et al., 2014). Whilst the exact mechanism by which Nsp3-4-6 can trigger this event is still unknown, the investigation of fully assembled virions by cryo-ET by Klein and co-authors has allowed to shed light on the dynamics of DMVs assembly and fusion during virus budding. This study showed that DMVs content in RNA is relatively high and their role might involve concealment of RNA intermediates from the immune system (Klein et al., 2020). The authors were further able to prove that, similarly, to other viruses, SARS-CoV-2 DMVs can form multi-vesicular compartment through membrane fusion. Instances of multiple vesicles contained in one single outer membrane, referred as vesicle packets (VPs), were observed and in some cases, inner membrane fusion allowed inter-connection between vesicle packets (Klein et al., 2020). Although a role for multi-vesicular DMVs in virus budding has been suggested, this has still to be experimentally verified. Virus budding was observed to occur in regions with a high concentration of DMVs and the authors observed that S proteins alone are not sufficient for membrane curvature, hypothesizing that other proteins, such M, initiate this process (Klein et al., 2020). These findings provide great advance in understanding the fundamental stages of virus replication and assembly. Nevertheless, further investigation and molecular details are required to understand how the membrane curvature occurs and the role of M protein and, potentially of other proteins in this process.

The PDB IDs for the aforementioned structures of the Nsp3 macro and papain-like protease domains can be found in Table 1. Nonetheless, the structure of the full-length protein remains to be determined, and in particular it is not clear if the Nsp3 protein forms a large, defined structure, or if it is merely constituted of isolated “beads-on-a-string”-like domains.

Nsp3 plays a central role in viral replication, and therefore is an attractive target for therapeutics development. However, there are still a lot of uncharacterized domains, and the relevance to its interaction with other non-structural proteins remains to be elucidated. In particular, an effort to obtain structural information of Nsp3 in complex with other proteins could provide several drug targets to block viral replication in host cells.

**FIGURE 3:**
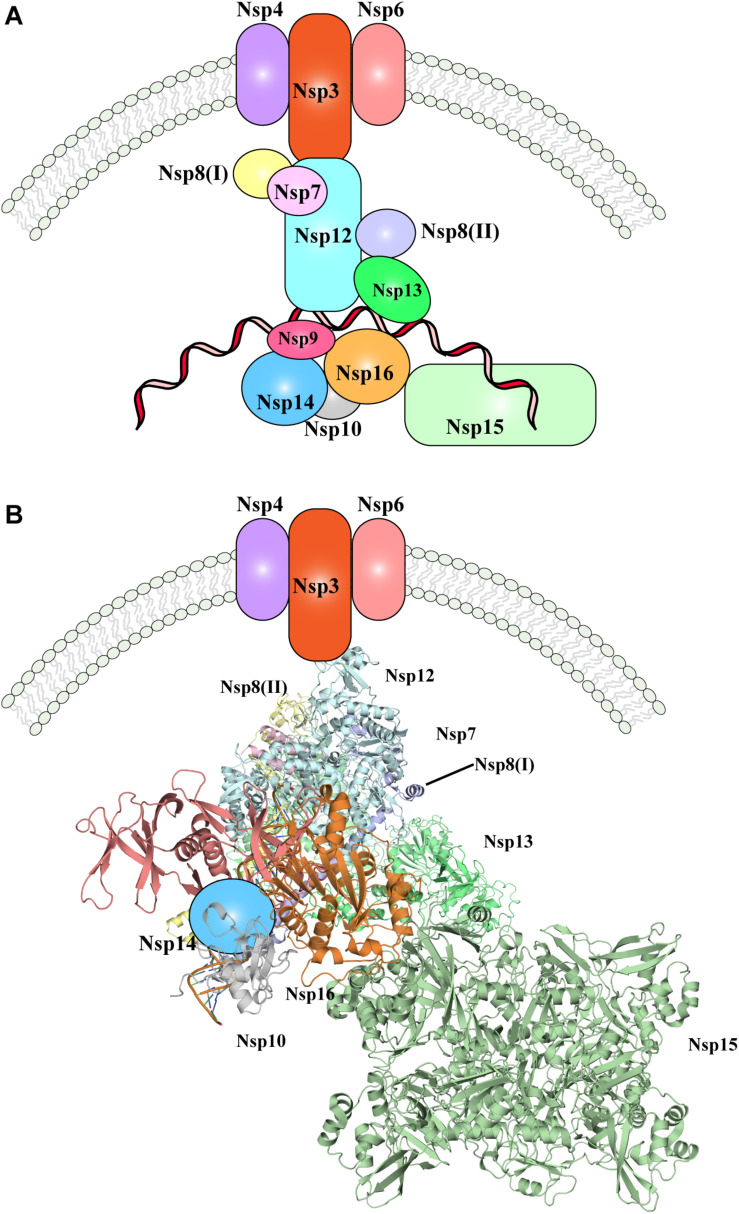
The SARS-CoV-2 replication-transcription complex. **(A)** Schematic representation of the non-structural proteins that were reported to intervene during the distinct steps of viral RNA replication. Endoplasmic reticulum rearrangements are performed by the Nsp3-5-6 subcomplex resulting in recruitment of the Nsp proteins, initiating RNA synthesis, proof-reading and capping. **(B)** Structural representation of the proteins involved in viral RNA replication, as shown in **(A)**. Where protein structures have been determined, cartoon models of the molecular structures are represented, whilst proteins with no structural details are depicted as colored shapes, as in part A. Relative positions and molecular contacts between Nsp proteins represented are based on the experimental evidence reviewed in this manuscript, while putative localization have been provided for those protein whose interaction network is still not determined. PDB IDs for the structures depicted in these figures are reported in Table 1.

Nevertheless, one of the most important pending questions is to understand how the whole replication-transcription complex elements cooperate and/or are hierarchically recruited during viral replication. In Figure 3, the available evidence of SARS-CoV-2 replication-transcription complex recruitment to date is represented. Putative localization and interactions networks have been presented based on the evidences reviewed in this manuscript.

Following cleavage by the proteases Nsp3 and Nsp5 (presumably still on the uncleaved full polypeptide, or carried over within virus particles), the individual Nsp proteins are produced. Nsp3-4-6 initiate ER rearrangements, which in turn recruits the remaining components of the RTC complex. Nsp12-7-8, together with Nsp13 are then likely responsible for production of novel viral RNA, and further modification and proof-reading is operated by Nsp10-14-16 complex (Figure 3). The role played by Nsp9 and Nsp15 within the RTC complex and the step at which their function is executed has not been fully elucidated, but given the interactions and co-localization of Nsp9 and Nsp15 with other components of the RTC complex, they are likely involved in RNA production or processing (Figure 3). Moreover, the N protein will be recruited *in situ* through interaction with Nsp3, however it remains to determine at which stage of the RNA replication its role is exerted.

While it is possible to propose a tentative model of the RTC role and functions within the infected host cells, many knowledge gaps are currently present that prevent to fully understand how SARS-CoV-2 replication works. For instance, it is not clear whether the recruitment of the RTC components and the exertion of their function is simultaneous or whether this is a timely coordinated process. In the latter case, it would be of great interest to determine the full hierarchy of Nsp proteins recruitment and the inter-dependence that exists between them. In particular, in the context of the interaction network of the Nsp proteins, their relative fold and orientation within the RTC complex should be determined, as well as a full characterization of the regions that are involved in protein-protein interactions and with the nascent RNA molecules. To date, Nsp1 and Nsp2 involvement in RTC complex or the existence of a mechanism that regulates and coordinates their activities with the RTC complex has not been reported and thus it is currently not clear where the role of Nsp1-2 falls within this preliminary putative model.

Similarly, the reported interactions of Nsp3 with Orf3a, Orf7a and Orf9b (von Brunn et al., 2007; Neuman et al., 2008; Angelini et al., 2013; Hagemeijer et al., 2014; Lei et al., 2018) also pose the question on whether these interactions are conserved in SARS-CoV-2 and whether other accessory proteins are involved in the various steps of the RTC assembly and mechanism.

Efforts to answer these questions could provide a comprehensive molecular dissection of the interplay between the SARS-CoV-2 proteins during viral replication and greatly improve the understanding of RTC complex function in its physiological context by taking into account also the supportive role that the accessory proteins can provide. It will also provide new avenues for targeting these interactions by antiviral drugs.

### Nsp1, Nsp2, Nsp5

Nsp1 displays high sequence divergence amongst the *Betacoronavirus* genus. Nevertheless, numerous reports on distinct viruses have highlighted that its function is conserved, despite the relatively low sequence similarity (Narayanan et al., 2015). In SARS-CoV, Nsp1 abrogates translation of the host cell genes, which may in turn promote enhanced translation of viral proteins (Lokugamage et al., 2012). This is achieved through two strategies; by binding of Nsp1 to the 40S ribosomal subunit and inhibiting mRNA translation at different stages (Kamitani et al., 2009; Lokugamage et al., 2012); and by endonucleolytic RNA cleavage in the 5′-UTR of host mRNAs (Kamitani et al., 2009). The translational inhibition does not apply to the viral mRNAs, however how this is achieved is unknown, but might implicate the interaction of Nsp1 with the 5′-UTR region of the viral mRNAs (Huang et al., 2011). Translational inhibition also determines lack of an efficient IFN-dependent antiviral signaling and, consequently, of efficient innate immune response (Narayanan et al., 2008). Importantly, amino acids that are crucial for Nsp1 functions have been identified and suggested as potential targets for therapy or generation of attenuated virus for vaccine development (Narayanan et al., 2008, 2015; Lokugamage et al., 2012).

Recent studies have confirmed that Nsp1 from SARS-CoV-2 plays the same roles as its counterpart in SARS-CoV during virus infection (Schubert et al., 2020; Thoms et al., 2020). In the study by Thoms et al. (2020) a complex between purified Nsp1 and 40S ribosomal subunits was reconstituted i*n vitro* by cryo-EM. The authors found two densities that they identified as two helices from Nsp1 C-terminus. These helices are localized inside the ribosomal mRNA entry channel, blocking mRNA accommodation and thereby shutting down mRNA translation (Thoms et al., 2020). The authors further identified a globular density, which may represent the N-terminal domain of Nsp1, but were unable to confirm it (Thoms et al., 2020). Importantly, Nsp1 C-terminal densities were also observed when the protein was co-purified with 40S and 80S ribosomal complexes *in vivo*. Structural data from these isolated complexes highlighted that Nsp1-40S and Nsp1-80S complexes could be divided in different populations, representing different intermediate states of the translation initiation process. In all these distinct intermediates, Nsp1 was found to bind in the position and conformation to that observed in the *in vitro* complex, confirming its role as inhibitor of translation initiation (Thoms et al., 2020). These findings were confirmed in an independently resolved structure of Nsp1 in complex with several ribosomal components (Schubert et al., 2020).

Much less is known instead about Nsp2. A study on the SARS-CoV Nsp2 protein has shown the ability of this protein to interact with prohibitin 1 and prohibitin 2 (Cornillez-Ty et al., 2009), however, this protein was found to be dispensable for virulence of several coronaviruses (Graham et al., 2005). Prohibitin proteins play a role in apoptosis regulation, mitochondrial functions and cell cycle regulation (Wang et al., 1999; Peng et al., 2015; Signorile et al., 2019). These findings imply that Nsp2, similarly, to Nsp1, may contribute to viral replication by arresting normal host cell functions. Whilst there are no available structures for Nsp2, a bioinformatic approach allowed to predict the presence of 4 putative transmembrane helices and a region that displays structural similarity to the endosome−associated protein of the avian infectious bronchitis virus (Cornillez-Ty et al., 2009). This latter region contains an amino acid substitution, compared to SARS-CoV, which might stabilize the protein and contribute to the higher infectivity of the virus (Angeletti et al., 2020).

Nsp5 is the main protease and, together with the papain-like protease domain in Nsp3, is responsible of cleavage of Orf1ab polypeptides and release of the mature Nsp proteins (Mousavizadeh and Ghasemi, 2020). Proteases and Nsp5 in particular have been identified as one of the most attractive drug targets to treat coronaviruses infections (Anand et al., 2003). Indeed inhibition of the Nsp5-mediated cleavage would prevent Nsp proteins productions and thus, prevent viral replication. For this reason, since the start of the SARS-CoV-2 pandemic, many studies have focused on determining the structure of Nsp5, alone or in complex with potential inhibitors, and have allowed to identify a plethora of compounds that are able to antagonize Nsp5 activity, providing the molecular mechanism behind the inhibition (Jin et al., 2020a, b; Zhang et al., 2020b). The chymotrypsin-like and 3C protease-like domains (domain I and II, respectively) both adopt a six-strand antiparallel β barrell fold. Domain III shows a globular fold that consists of a group of five helices. Interaction between two domains III from two distinct Nsp5 protomers is responsible of modulating the dimerization between their respective domain I and II. SARS-CoV-2 Nsp5 dimerises with the same affinity as its SARS-CoV counterpart (Zhang et al., 2020b). Dimerization of Nsp5 protomers ultimately results in the formation of a substrate-binding catalytic site, where Cys^145^ and His^41^ from each protomer constitute the catalytic residues (Zhang et al., 2020b). These reports demonstrated that the overall structure of SARS-CoV-2 Nsp5 is similar to SARS-CoV Nsp5, albeit some minor structural differences were found. In particular, a hydrogen bond between Thr^285^ residues of the two domains III of SARS-CoV Nsp5 was observed (Zhang et al., 2020b). In SARS-CoV2 Nsp5, Thr^285^ is replaced by an Alanine residue, thereby abolishing the hydrogen bond. Absence of this bond allows the domain III from each SARS-CoV2 Nsp5 to come closer contact, but only causes a slight increase in catalytic efficiency, compared to SARS-CoV (Zhang et al., 2020b).

Interestingly, several promising drugs were already designed for other purposes, including targeting of SARS-CoV and seem to be successfully repurposed for Nsp5 inhibition (Jin et al., 2020a, b; Zhang et al., 2020a, b). For most of such compounds, these structures revealed that the molecules act as competitive inhibitors, binding in the substrate-binding pocket (Jin et al., 2020a, b; Zhang et al., 2020b).

## Accessory Proteins

Severe acute respiratory syndrome coronavirus 2 accessory proteins are generally less well characterized, and their role in infection is mostly not understood. Accessory proteins display the highest degree of variability, even within very closely related coronaviruses (Wu A. et al., 2020). Indeed, Orf10 is exclusively present in SARS-CoV-2 whilst it is not encoded by the closely related SARS-CoV. Similarly, SARS-CoV encodes two Orfs, Orf8a and Orf8b, in place of the single Orf8 that is conversely found in SARS-CoV-2 (Nguyen et al., 2020). Investigation of the role of the accessory proteins and the different pathways they influence could allow to pin-point those proteins responsible for instance, of the different infectivity or mortality of SARS-CoV and SARS-CoV-2 viruses.

Orf3a displays high conservation in the *Betacoronavirus* subgenus *Sarbecovirus*, which includes SARS-CoV and SARS-CoV-2 (Kern et al., 2020). In SARS-CoV, Orf3a was shown to form an ion channel likely causing a calcium influx, involved in increased formation of DMVs (Lu et al., 2009). This pore shows modest selectivity towards K^+^ and Ca^2+^ over Na^+^ *in vitro*, however, the ion actually transported *in vivo* is not known. The structure of SARS-CoV-2 Orf3a in its inactive or ‘closed’ conformation has been solved with cryo-EM, highlighting that this protein adopts a novel fold, forming a large and bifurcated channel that is connected to the cytoplasm through a large cavity (Kern et al., 2020). Orf3a was found to determine apoptosis and in SARS-CoV the pro-apoptotic activity of Orf3a has been associated to its channel-forming activity (Chan et al., 2009; Ren et al., 2020).

Orf3a can additionally interact with the structural proteins N, M and S (Tan, 2005), supporting a role of Orf3a in virus budding. These observations are consistent with the reported interaction between Orf3a and Nsp3 (von Brunn et al., 2007), which initiates DMVs formation.

Nsp3 also interacts with Orf9b, suggesting that the role of these three proteins might be connected. Orf9b is a E3 ubiquitin ligase, and evidences have shown that it promotes the ubiquitination and proteasomal degradation of dynamin-like protein 1 (Box 2) (Kamerkar et al., 2018). Dynamin-like protein 1 promotes mitochondrial fission in physiological conditions. Orf9b-mediated degradation of dynamin-like protein 1 may in turn lead to elongation of mitochondria, ultimately compromising the association between mitochondria and ER, a crucial step for IFN production and signaling during innate immune response (Chatel-Chaix et al., 2016; Kim et al., 2018). Furthermore, it hinders the cell IFN response by triggering the degradation of MAVS, TRAF3 and TRAF6 (Shi et al., 2014; Sa Ribero et al., 2020). A structure for SARS-CoV-2 Orf9b has been deposited in PDB (Table 1), where it shows high conservation with SARS-CoV Orf9b dimeric, β-sheet-rich, structure (Meier et al., 2006). Additionally, a central, lipid-binding pocket was notably observed and it was suggested to mediate ORF9b localization in proximity of ER/Golgi complex (Meier et al., 2006). Furthermore, Orf9b was proposed to be involved in the interaction with Nsp3, N and S proteins (von Brunn et al., 2007; Lu et al., 2009). Binding to the ER/Golgi complex membrane, through its lipid binding pocket, would provide Orf9b with an ideal localization to accomplish these interactions and would suggest a role for this protein in virion assembly.

Despite the currently available evidence, the exact role(s) of Orf9b is not fully understood. Specifically, it is not known if the Orf9b interactions are all necessary for its Ubiquitin Ligase activity, or whether Orf9b interactions with other SARS-CoV-2 proteins indicate that it plays multiple roles in the virus cycle. Structural characterization of these sub-complexes could provide the necessary information to understand the role of Orf9b.

Like Orf3a, evidence suggests that Orf7a also might play a role in virus budding and release. In SARS-CoV, ORF7a enables virion release, by preventing the antiviral activity of the bone marrow stromal antigen 2 (BST-2/tetherin), which tethers budding virions to the host cell (Taylor et al., 2015). In agreement with this, Orf7a was also shown to interact with Nsp3 (Neuman et al., 2008). In SARS-CoV, Orf3a and Orf7a were additionally found to localize at the Golgi-endoplasmic reticulum intermediary compartment, with Nsp3 (von Brunn et al., 2007; Taylor et al., 2015), where Orf7a can additionally interact with Orf3a, which in turn interacts with M, N and S proteins (Tan, 2005). However, recent observation in SARS-CoV-2 found that Orf3a is mainly localized at the plasma membrane (Kern et al., 2020; Ren et al., 2020). The structure of SARS-CoV-2 Orf7a has been solved (PDB ID: 6W37, Table 1), and it shows high degree of conservation with the structure of SARS-CoV Orf7a, revealing that this protein possesses an Ig fold (Nelson et al., 2005). It also possesses a predicted C-terminal TM helix.

Orf8 has newly been described as a paralog of Orf7a, thought to have evolved via gene duplication. It also possesses an Ig-like fold, but Orf8 lacks the TM helix, and has an additional large insertion between its third and fourth strand. A homology model of Orf8, based on the solved Orf7a structure has suggested that this insertion in Orf8 would lead to an enhancement of the putative peptide-ligand binding activity that was reported for Orf7a (Tan et al., 2020). Orf8 binds to MHC-I, leading to decreased antigen presentation, whereas this was not observed for Orf8a and 8b. Orf8-mediated inhibition of MHC-I antigen presentation ultimately leads to inefficient viral clearing (Zhang et al., 2020c). Additionally, Orf8 was found to inhibit IFN-I signaling pathway, likely through downregulation of the Interferon Stimulation Response Element (ISRE) (Li J.-Y. et al., 2020). In SARS-CoV, Orf8b was shown to interact with Orf7a, M and E proteins (Keng and Tan, 2009), leaving open the possibility that it could additionally cooperate in DMVs formation and virus budding. It is not known if SARS-CoV-2 Orf8 possesses a similar activity. Future efforts should address this question and further clarify the basis of the different localization observed for Orf3a in SARS-CoV and SARS-CoV-2. Answers to the latter questions will further allow to shed light on whether Orf3a interaction with Orf7a and Nsp3 is retained in SARS-CoV-2 and in this case, to work towards determination of the molecular details of the interactions between Orf3a, Orf7a with Nsp3 and the other proteins found at the DMVs interface, as well as interactions with the structural proteins during virus budding, to establish the contacts that are required for virus budding and release.

Orf7b is predicted to be an integral membrane protein and its single TMH was found to be sufficient to direct localization to the Golgi complex in SARS-CoV (Schaecher et al., 2008). Furthermore, Orf7a and Orf7b favor apoptotic activity of infected cells, although their role is dispensable for SARS-CoV infections in Vero African green monkey kidney cells, CaLu-3 human lung adenocarcinoma cells and CaCo-2 human colorectal carcinoma cells (Schaecher et al., 2007). To date, research efforts focused on SARS-CoV-2 investigations have not provided many details on Orf7b functions. Given the high sequence similarity between Orf7b in the two closely related viruses, it likely that its role will be conserved. Nevertheless, a structure Orf7b from either SARS-CoV and SARS-CoV-2 has not yet been determined.

Orf3b has been recently identified as one of the accessory proteins of SARS-CoV-2 that downregulates IFN-I signaling (Konno et al., 2020) (Box 3). Importantly, SARS-CoV-2 Orf3b ability to antagonize IFN-I is significantly higher than its homologue in SARS-CoV (Konno et al., 2020), which may play a role in the higher transmission rate. This function is determined by the C-terminus of Orf3b, and specifically correlates with length of this region (Konno et al., 2020). To date, there are no studies that report a structure for Orf3b. Determination of structural details could shed light onto the nature of its fold, facilitating identification of proteins with structural similarities, which may in turn provide a better understanding on the putative roles of this protein and locate sites to target.

**BOX 3. SARS-CoV-2 manipulation of the Interferon response pathway.**

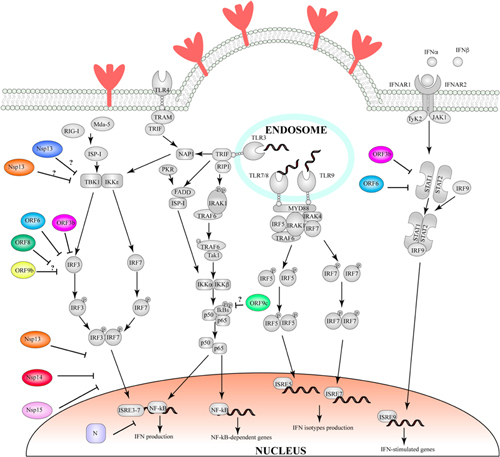

**Figure |** Schematic representation of SARS-CoV-2 proteins and their involvement in IFN hijacking. Proteins involved in IFN production pathways were represented in gray, while SARS-CoV-2 proteins are represented in color. The stage of IFN pathways which non-structural or accessory proteins are predicted to interfere with, is based on the reported studies cited within the box and the main text.

Interferons are a class of cytokines, central to the innate immune response (for extensive review of the pathway, see Muller et al., 1994; Samuel, 2001; Zhou et al., 2018). The release of IFN, a response to cell infection by a viral (or bacterial) pathogen, triggers downstream events to prevent their replication, such as suppression of viral transcription, translation and replication and prevention of entry of the virus (Samuel, 2001), which ultimately protect the host. Given the broad spectrum of IFN-dependent response, many viruses, including SARS-CoV-2, have evolved multiple effective strategies to subvert this defense (Spiegel et al., 2005; Hadjadj et al., 2020).

A number of SARS-CoV-2 proteins have been implicated in modulating the IFN response either at the level of IFN synthesis, downstream IFN-signaling, or both (Sa Ribero et al., 2020). Over-expression of Orf3b, Orf6 and N protein has been demonstrated to antagonize IFN (Yuen et al., 2020; Konno et al., 2020; Li J.-Y. et al., 2020). In SARS-CoV, these proteins were demonstrated to play a role in IFN-ß downregulation, via repression of the interferon regulatory factor-3 (IRF-3) (Kopecky-Bromberg et al., 2007). Hindering of this response results in enhanced viral replication, as IFN-ß stimulates the expression of antiviral and antiproliferative factors by neighboring cells, thereby limiting spread (Ivashkiv and Donlin, 2014). Orf6 inhibits IFN production through hindering of the of STAT1-nuclear complex formation (Frieman et al., 2007; Lei et al., 2020) by sequestering karyopherin α2 (KPNA2) and karyopherin ß1 (KPNB1) to the endoplasmic reticulum/Golgi interface, thereby preventing nuclear translocation of the messenger STAT1 and, consequently hampering the host defense against viral infection (McBride and Reich, 2003; Frieman et al., 2007).

Orf3b has also been shown to bind to karyopherins α3 and α4 (Veljkovic and Paessler, 2020). Interestingly, the same mechanism of IFN inhibition is shared by the Ebola virus, whereby VP24 binds karyopherins α1, α5 and α6 (Reid et al., 2007).

Alongside Orf6 and Orf3b, Orf8 and N have also been shown to inhibit IFN-I and the NF-κB response (Li J.-Y. et al., 2020). Both Orf6 and Orf8 reduce expression of the Interferon Stimulation Response Element (ISRE), whereas N has been proposed to use an alternative mode of IFN downregulation (Li J.-Y. et al., 2020): it was shown to interact with stress granule proteins G3BP1, G3BP2 and La-related protein 1 (LARP1) (Gordon et al., 2020). G3BP1 and G3BP2 upregulate and allow accumulation of interferon-stimulated genes (ISG), whereas the role of LARP1 in ISG regulation has been object of controversial reports (Alam and Kennedy, 2019; Kim et al., 2019; Berman et al., 2020).

More recently, Nsp13, Nsp14 and Nsp15 of SARS-CoV-2 have also been implicated in the inhibition of nuclear localization of IRF-3 (Yuen et al., 2020; Gordon et al., 2020). Putative interactions between Orf9c and multiple regulators of Iκß kinase, an upstream kinase of NF-κB which activates IRF-3, have also been reported (Gordon et al., 2020). Furthermore, an interaction between Orf9b and the adaptor protein TOMM70, required for IRF-3 activation, was also observed (Gordon et al., 2020). Nevertheless, the physiological relevance of these interactions has yet to be determined. Finally, the papain-like domain on Nsp3 was also found to hamper the IFN-I response by altering the normal ISG15-modifications pathway, through cleavage of ISG15 (Fu et al., 2020).

The sensitivity of SARS-CoV to IFN treatment has been known for many years (Cinatl et al., 2003). IFN-I is therefore an emerging candidate for COVID-19 treatment, as IFN-α and IFN-ß are also able to inhibit SARS-CoV-2 replication (Hensley et al., 2004; Clementi et al., 2020; Felgenhauer et al., 2020). Yet to this date, we do not have any structural information on the mechanisms by which the virus overcomes this effect. Such data will be necessary to exploit the interferon response in the combat against COVID19.

Similar to Orf3b, Orf6 also down-regulates interferon signaling (Yuen et al., 2020). Previous immunofluorescence studies performed for SARS-CoV demonstrated that Orf6 achieves IFN-I antagonism by disrupting formation of STAT1 complex, through sequestering its components to the endoplasmic reticulum/Golgi complex (Boxes 2, 3) (Frieman et al., 2007; Lei et al., 2020). Whilst this mode of action needs to be confirmed for SARS-CoV-2, an additional interaction between Orf6 and an mRNA export factor induced by IFN-I, NUP98 RAE1, has been reported (Gordon et al., 2020). Therefore, identifying the key binding interactions between Orf6 and its binding partners by solving the complex structure could be crucial for understanding the mechanism of inhibition employed and resultant pathogenesis. In addition to IFN-I antagonism, it has been suggested that Orf6 could play a role in viral replication: In SARS-CoV, Orf6 was found to co-localize and interact with Nsp8 (Kumar et al., 2007). It is currently unclear whether Orf6 is involved in RNA synthesis or whether its interaction with Nsp8 might play a modulating role on the activity of the replication-transcription complex. Furthermore, it still needs to be investigated whether the interaction between Orf6 and Nsp8 and its physiological role in viral replication are conserved in SARS-CoV-2.

Finally, very few information are available for Orf9c and Orf10. SARS-CoV-2 Orf9c is encoded within the nucleocapsid gene, similarly, to Orf9b. Evidence in SARS-CoV suggests that it interacts with human NLRX1, F2RL1, NDFIP2, proteins, which in turn are associated with NF-kB signaling pathway (Gordon et al., 2020). Orf9c can further influence the modulation of IkB kinase (Gordon et al., 2020), thereby suggesting a role of this protein in modulation of inflammation.

Orf10 is exclusively found in SARS-CoV-2 and its use has been suggested as a strategy to identify ongoing COVID-19 infections (Koyama et al., 2020). Available details about Orf10 are very few, however, a recent analysis of the interactome of SARS-CoV-2 proteins has interestingly pointed out that that Orf10 interacts with the Cullin2 RING E3 ligase complex, in particular with the ZYG11B adapter (Gordon et al., 2020) (Box 2). The physiological role and mechanistic of this interaction is not known, but it suggests that as many other viral proteins, Orf10 is involved in hijacking the ubiquitin-proteasome complex in order to favor viral replication and inhibit the antiviral host response, with a mechanism that is yet to be determined.

## Conclusive Remarks

SARS-CoV-2 first appeared in China in 2019 and has quickly become a major global threat to public health. Despite the impressive amount of research efforts that have been devoted to fully characterize the mode of infection of this novel virus and determine the details of its mechanism of hijacking on host factors, a specific treatment with high rate of success has not been yet found. Similarly, a clinically approved vaccine has not been developed yet, despite the great advances that have been made in the characterization of SARS-CoV-2 entry into host cells.

In this review we have summarized the current state of knowledge of the structural studies that have provided detailed molecular details of some of the SARS-COV-2 proteins and the basis of their biological functions during the viral infection cycle. Furthermore, several of the reported studies within this review have employed X-ray crystallography and cryo-EM approaches to not only determine the mode of action of SARS-COV-2 proteins in their physiological context but to additionally determine the basis of inhibition of their activity operated by potential inhibitors that may be developed for treatment of COVID-19.

Nevertheless, there are many aspects of the distinct phases of SARS-CoV-2 infection cycle that are not well characterized. For instance, despite the high number of structures now available for the SARS-CoV-2 S protein in its open or closed state or in complex with the ACE2 receptor, it still remains to be understood how the TMDs of the S protein effectively contribute to the structural rearrangements that are coupled with virus entry. One of the main challenges still to be addressed at the structural level, is to obtain high-resolution details of membrane domains from the other structural proteins (M and E).

Similarly, many structures of the replication-transcription complex have been determined, providing new insights into the mechanism of RNA synthesis, the basis of the proofreading activity operated by Nsp13 and the regulatory role of the conformational changes of Nsp7 and Nsp8. Nevertheless, multiple studies in SARS-CoV have indicated that accessory proteins may intervene at different stages of the replication process or in the previous phase of DMVs formation (Kumar et al., 2007; von Brunn et al., 2007; Neuman et al., 2008; Angelini et al., 2013; Hagemeijer et al., 2014; Lei et al., 2018). However, the molecular basis for this is largely not understood.

Research effort to date have provided molecular details of several subcomplexes involved in viral entry, replication and budding. However, one of the current limitations in fully understanding these processes is lack of structural and functional characterization of the full protein complexes, protein interaction networks and its hierarchy, involved in the main steps of SARS-CoV-2 infection. Integrating the available structural data on subcomplexes involved in viral replication with additional functional and biochemical studies will provide information on crucial protein-protein interactions within the full complexes, including transient interactions that play a modulatory role or mediate distinct phases of infection. In particular, accessory proteins may play multifaceted role in infection, and require further characterization. Ultimately, a more comprehensive knowledge of SARS-CoV-2 infection that these additional studies would provide will allow a more effective and specific structure-guided design of vaccination and therapeutic strategies. Structural biologists, like Wayne Gretzky, “need to skate to where the puck is going to be, not to where the puck has been”.

## Author Contributions

JRCB and GM planned the overall structure of the review. GM wrote the manuscript and made the figures, with help from RJF and SLML-F for the accessory proteins section, and with edits from JRCB. All authors contributed to the article and approved the submitted version.

## Conflict of Interest

The authors declare that the research was conducted in the absence of any commercial or financial relationships that could be construed as a potential conflict of interest.
